# Randomized phase II trial of autologous dendritic cell vaccines versus autologous tumor cell vaccines in metastatic melanoma: 5-year follow up and additional analyses

**DOI:** 10.1186/s40425-018-0330-1

**Published:** 2018-03-06

**Authors:** Robert O. Dillman, Andrew N. Cornforth, Gabriel I. Nistor, Edward F. McClay, Thomas T. Amatruda, Carol Depriest

**Affiliations:** 1 Hoag Cancer Institute, Newport Beach, CA 92660 USA; 2 AIVITA Biomedical, Inc., Irvine, CA USA; 3 TCR2 Therapeutics, Cambridge, MA USA; 4 California Cancer Associates for Research and Excellence (cCARE), Institute for Melanoma Research & Education, Encinitas, CA USA; 5 Minnesota Oncology, Fridley, MN USA; 6 Franklin, TN USA

**Keywords:** Metastatic melanoma, Patient-specific vaccines, Dendritic-cell vaccines, Tumor-cell vaccines, Autologous tumor cell lines

## Abstract

**Background:**

Despite improved survival following checkpoint inhibitors, there is still a potential role for anti-cancer therapeutic vaccines. Because of biological heterogeneity and neoantigens resulting from each patient’s mutanome, autologous tumor may be the best source of tumor-associated antigens (TAA) for vaccines. Ex vivo loading of autologous dendritic cells with TAA may be associated with superior clinical outcome compared to injecting irradiated autologous tumor cells. We conducted a randomized phase II trial to compare autologous tumor cell vaccines (TCV) and autologous dendritic cell vaccines (DCV) loaded with autologous TAA.

**Methods:**

Short-term autologous tumor cell lines were established from metastatic tumor. Vaccines were admixed with 500 micrograms of GM-CSF and injected weekly for 3 weeks, then at weeks 8, 12,16, 20, and 24. The primary endpoint was overall survival. Secondary objectives were identification of adverse events, and results of delayed type hypersensitivity (DTH) reactions to intradermal tumor cell injections.

**Results:**

Forty-two patients were randomized. All were followed from randomization until death or for five years; none were lost to follow-up. DCV was associated with longer survival: median 43.4 versus 20.5 months (95% CI, 18.6 to > 60 versus 9.3 to 32.3 months) and a 70% reduction in the risk of death (hazard ratio = 0.304, *p* = 0.0053, 95% CI, 0.131 to 0.702). Tumor DTH reactions were neither prognostic nor predictive. The most common treatment-related adverse events were mild to moderate local injection site reactions and flu-like symptoms; but grade 2 treatment-related adverse events were more frequent with TCV. Serum marker analyses at week-0 and week-4 showed that serum markers were similar at baseline in each arm, but differed after vaccination.

**Conclusions:**

This is the only human clinical trial comparing DCV and TCV as platforms for autologous TAA presentation. DCV was associated with minimal toxicity and long-term survival in patients with metastatic melanoma. DTH to autologous tumor cells was neither prognostic for survival nor predictive of benefit for either vaccine.

**Trial registration:**

Clinical trials.gov NCT00948480 retrospectively registered 28 July 2009.

**Electronic supplementary material:**

The online version of this article (10.1186/s40425-018-0330-1) contains supplementary material, which is available to authorized users.

## Background

### Relevance of therapeutic vaccine research

During the past decade introduction of new therapies has been associated with increased survival for patients with metastatic melanoma [1]. These have included oral enzyme inhibitors of signal-transduction driver pathways [2, 3], and monoclonal antibodies that block immune checkpoint inhibitors [4–6]. However, even with combination checkpoint inhibitors as initial therapy, the 3-year survival rates are less than 60% [7]. Therapeutic vaccines might add to the survival benefit of such patients. First, animal models suggest that anti-tumor vaccines and checkpoint inhibitors are complementary therapies [8, 9]. Second, the mechanism of action for the anti-programmed death-1 (PD1) checkpoint inhibitors is blocking the suppression of an existing immune response, but many patients show no evidence of an existing immune response in their tumors [10, 11]. Third, therapeutic vaccines have been associated with survival benefit in some patients [12, 13]. Fourth, dendritic cell vaccines can induce or enhance immune responses to patient-specific neoantigens [14]. For these and other reasons, vaccine research is still relevant for melanoma treatment [15].

### Autologous tumor cells as sources of tumor-associated antigens

Because of biological heterogeneity and neoantigens resulting from each mutanome [16, 17], autologous tumor may be the best source of tumor-associated antigens (TAA) for vaccines. Short-term tumor cell lines derived from metastatic lesions is one source of TAA that could be used for patient-specific vaccines [18, 19]. They can express all TAA, including unique patient-specific TAA expressed only on self-renewing, proliferating autologous tumor cells that may represent tumor initiating stem cells and/or early progenitor cells. Using tumor cells from a short-term cell line assures lack of contamination with viable normal cells or immune suppressor cells such as regulatory T cells, or myeloid derived suppressor cells.

### Whole tumor cell vaccines versus dendritic cell vaccines

Two potential therapeutic approaches with short-term tumor cell lines include (1) injecting irradiated tumor cells (ITC) in a manner similar to many anti-viral vaccines, and then relying on endogenous antigen-presenting cells to process TAA and induce anti-tumor immune responses; and (2) loading antigens from such cells ex vivo into autologous dendritic cells (DC) and vaccinating with these cells. Patient-specific tumor cell vaccines (TCV) derived from autologous short-term tumor cell lines were used to treat 74 metastatic melanoma patients. TCV was well-tolerated, associated with an objective response rate (ORR) of 9%, progression free survival (PFS) of 4.4 months, and when all patients were followed to death or for 5 years from enrollment with none lost to follow-up, median overall survival (OS) was 20.5 months, and 5-year OS 28% [20]. Subsequently 54 metastatic melanoma patients were treated with patient-specific dendritic cell vaccines (DCV) consisting of autologous dendritic cells (DC) loaded with TAA by incubating DC with 10 million ITC derived from autologous tumor cell lines. DCV was well-tolerated, associated with an ORR of 0%, PFS of 4.2 months, median OS greater than 5 years, and projected 5-year OS of 54% [21]. When all patients were followed to death or for 5 years from enrollment with none lost to follow-up, 5-year OS was 50%.

### Significance of current report

A randomized trial (MACVAC) to compare these two approaches was initiated in October 2007, and is still the only human trial that has addressed whether a DCV is superior to a TCV. The trial was stopped prematurely due to discontinuation of financial support by the sponsoring hospital in April 2011. By then 42 patients had been randomized; all had initiated treatment per randomization assignment. Preliminary results were reported at a time when all patients had completed therapy; so minimum follow up was six months, median follow up was less than two years, maximum follow up less than four years, and 21 patients were deceased. OS was better in the DCV arm with projected 2-year survival rates of 72% versus 31% (*p* = 0.007) [22]. New information in the current report includes: (1) survival analysis performed after all patients had been followed for five years or until death, (2) details regarding patient characteristics and treatments administered before and after participation in this study, (3) results of a multivariate Cox regression analysis and hazards model, (4) survival results by treatment arm in various subsets of patients, (45) comparative results of delayed type hypersensitivity (DTH) reactions to autologous ITC, (6) comparison of adverse events by treatment arm, (7) comparison of serum cytokine results before and after vaccination, (8) and additional analyses regarding the feasibility of establishing tumor cell lines.

## Methods

### Design

This was designed as an open-label, randomized trial to compare autologous DCV to autologous TCV. The primary endpoint was overall survival. Secondary objectives were identification of adverse events, and results of delayed type hypersensitivity (DTH) reactions to intradermal tumor cell injections.

#### Autologous tumor cell lines

Surgically resected metastatic tumors were submitted on behalf of melanoma patients classified as recurrent stage 3 or distant stage 4. Tumors were mechanically and enzymatically dissociated into single-cell suspensions and grown in tissue culture as previously described [18, 22, 23]. The patient’s managing physician was notified once a cell line was successful. These cell cultures were the source of ITC used for autologous tumor DTH tests, for TCV, and for co-incubation with DC to create DCV.

#### Autologous dendritic cells

DC were derived from peripheral blood mononuclear cells obtained during a single leukapheresis procedure and monocytes were separated (Elutra® Cell Separation System, CaridianBCT, Lakewood, CO.). Monocytes were differentiated into DC over six days in the presence of granulocyte-macrophage colony stimulating factor (GM-CSF) and interleukin-4 as previously described [21, 22].

#### Investigational products

TCV consisted of ITC derived from the patient’s autologous tumor cell line as previously described [21–23]. Each dose contained about 10 million ITC.

DCV consisted of autologous DC that were incubated overnight with 10 million ITC for phagocytosis and antigen loading as previously described [21, 22]. The final product averaged 10 to 15 million cells per dose, but ranged from 3 to 30 million per dose among different patients and included residual ITC in many samples. There was substantial inter-patient variation in average dose, but little intra-patient variation in doses.

#### Patients

Eligibility criteria for the MACVAC trial were previously described [22]. Key eligibility criteria for randomization were: (1) availability of short-term autologous tumor cell line, (2) referral by the managing physician for vaccine therapy, (3) willingness to travel to Newport Beach, California for treatment, and (4) Karnofsky performance status ≥ 70. Patients with brain metastases were eligible if they had been treated with expectation of successful control.

#### Randomization

Patients were stratified by whether their most advanced stage of disease was 3 or 4 [24], and by whether they had measurable disease at the time of randomization [25]. They were then randomized 1:1 without blocking. Patients randomized to TCV were able to undergo DTH testing the next day and start treatment the following week. Patients randomized to DCV underwent a leukapheresis procedure the same or following day and could begin DTH testing and treatment about four weeks later.

#### Treatment schedule

At the time of each treatment, a cryopreserved vial of TCV or DCV was thawed and suspended in 500 micrograms of GM-CSF and injected within five hours of thawing. Subcutaneous injections were administered during weeks 1, 2, 3, 8, 12, 16, 20 and 24, as in earlier trials [21, 22]. Concurrent anti-cancer therapy was not allowed, but there was a provision by which patients could interrupt vaccine therapy in order to pursue another therapy because of progressive disease, and then when that therapy was completed, finish the remaining vaccine doses.

#### Follow up information

After completion of vaccine injections, patients were followed in person or by telecommunications every three months to collect information regarding administration of additional anti-cancer therapies, any new adverse events (AE) that might be attributable to vaccine treatment, approximate date of disease progression, or date and cause of death if deceased.

#### End points

##### Survival

The primary endpoint was OS from the date of randomization per intent-to treat to date of death. Managing physicians and clinical trial staff identified the first site of disease progression and a date of disease progression to estimate PFS.

### Delayed type hypersensitivity (DTH) to autologous tumor cells

One week before starting either vaccine, patients were administered an intradermal skin test of 1 million ITC. Within 48 to 72 h the test was interpreted by nursing staff as negative (no induration), weakly positive (5 to 9 mm induration), or positive (≥ 1 cm induration). The test and interpretation was repeated one week after the three weekly injections had been administered.

### Safety

AE were assessed at each visit for a vaccine injection, and four weeks after the last injection. AEs and serious AEs (SAEs) were classified and graded 0 to 5 per National Cancer Institute Common Toxicity Criteria for Adverse Events (NCI-CTCAE) version 3.0.

#### Serum markers

Cryopreserved serum samples (200 μl) from week-0 and week-4 were sent to Raybiotech, Inc. (Norcross, GA) for human cytokine protein array screening for the 110 different proteins then available in their Quantibody® Cytokine Array, which utilizes a validated, quantitative, multiplex enzyme-linked immunosorbent assay (ELISA). In order to normalize the results of various assays, week-0 and week-4 values for various assays were expressed as differences above or below the mean values for three normal controls.

#### Statistical analysis

Survival curves were generated using the method of Kaplan and Meier and compared using log rank tests (Mantel-Haenszel and Gehan’s Wilcoxon). The Cox regression model and the Wald test were used to estimate the hazard ratio associated with treatment and to identify the significance of potential prognostic factors and their impact on treatment differences. Variables associated with 3-year survival were explored by multiple univariate analyses. Proportions were compared using the Pearson Chi Square or Fisher’s Exact Test. Means were compared using the Student T-test. Nonparametric means were compared using the Mann-Whitney test.

## Results

### Establishing tumor cell lines

As previously reported median time from tissue harvest to successful cell line was 3.1 months [22], but the range was from 27 to 224 days. The sites of tumor collection were lymph nodes (*n* = 21), cutaneous or subcutaneous tissue (*n* = 11), lung (*n* = 3), breast (*n* = 2), and one each from brain, omentum, liver, spleen, and one with multiple sites. The median tumor weight was 2.3 g (range < 0.2 to 12.5). The cell line success rate was 46/79 (58%) for tumors ≥ 3 g compared to 30/108 (28%) for those < 3 g (*p* < 0.0001) (Additional file 1: Table S1). Among patients for whom cell lines were successful, 42/76 (55%) were referred for study enrollment. The median time to a successful cell culture was 81.0 days for both TCV and DCV; average time was 94.6 days (range17 to 237 days). There was no correlation between time to establish a cell line and survival.

Median time from tissue harvest to treatment was 3.0 months (range 3 to 23 months). While efforts were in progress to establish a cell line, disease progression occurred in 62% of patients who were eventually treated with DCV or TCV (Additional file 2: Table S2). Among patients with recurrent stage 3 at the time of tumor tissue acquisition, 9/18 were still NED at the time of randomization; another who had developed M1c disease after resection had a complete response to interleukin-2-based biochemotherapy. The other eight had progressed to detectable metastatic disease (7 measurable). Among 24 patients who had distant metastases at the time tissue was collected, only 5 were free of disease at randomization.

### Treatment assignment, patient characteristics and other therapies

All patients were treated per randomization assignment (Additional file 3: Figure S1). Patient baseline characteristics at baseline are shown in Table 1. None of the specific individual characteristics differed significantly between the arms. There were no differences in therapies given previous or subsequent to DCV or TCV (Table 2). Patients were treated during November 2007 to August 2011 before widespread use of BRAF/MEK enzyme inhibitors, and the anti-cytotoxic T lymphocyte-4 (CTLA-4) and anti-programmed death-1 (PD-1) monoclonal antibody checkpoint inhibitors. The first FDA approvals of these agents were 2010 for ipilimumab, 2011 for vemurafinib, and 2014 for the anti-PD-1 inhibitors pembrolizumab and nivolumab.

**Table 1:** Baseline characteristics of patients and by treatment arm

Characteristic	All (*n* = 42)	TCV (*n* = 24)	DCV (*N* = 18)	*P*-value
Age ≥ 60 years	20 (48%)	12 (50%)	8 (44%)	0.72
# Male	27 (64%)	16 (67%)	11 (61%)	0.71
# from out of state	16 (38%)	9 (38%)	7 (39%)	0.93
KPS = 100%	21 (50%)	12 (50%)	9 (50%)	1.00
↑LDH at randomization	11 (26%)	4 (17%)	7 (39%)	0.16
Highest stage =4	33 (79%)	17 (71%)	16 (89%)	0.16
Prior brain metastases	10 (24%)	6 (25%)	4 (22%)	1.00
Prior visceral metastases (non-CNS)	22 (52%)	14 (58%)	8 (44%)	0.45
Measurable Disease	17 (40%)	9 (38%)	8 (44%)	0.65
Detectable (not measurable)	10 (24%)	4 (17%)	6 (33%)	0.28
NED at randomization	15 (36%)	11 (46%)	4 (22%)	0.19
Stage 4 M1a at randomization	3 (7%)	1 (4%)	2 (11%)	0.57
Stage 4 M1b at randomization	9 (21%)	6 (25%)	3 (17%)	0.71
Stage 4 M1c at randomization	15 (36%)	6 (25%)	9 (50%)	0.094

*TCV* tumor cell vaccine, *DCV* dendritic cell vaccine, *KPS* Karnofsky Performance Status, *LDH* serum lactate dehydrogenase, *CNS* central nervous system, *NED* no evidence of disease, *M1a* metastatic disease soft tissue metastases only and normal LDH, *M1b* metastatic lung with or without soft tissue metastases, but no other visceral metastases and normal LDH, *M1c* metastases to visceral organs and/or elevated LDH

**Table 2:** Anti-melanoma therapy prior and subsequent to participation in the MACVAC trial. Therapies: overall and by treatment arm

	All (*n* = 42)	TCV (*n* = 24)	DCV (*N* = 18)	*P*-value
Previous therapy				
Surgeries Only	7 (17%)	3 (12%)	4 (22%)	0.44
Radiation Therapy (not brain)	17 (40%)	8 (33%)	9 (50%)	0.28
Brain Radiation Therapy	10 (24%)	6 (25%)	4 (22%)	1.00
Chemotherapy	23 (55%)	15 (62%)	8 (44%)	0.24
Interleukin-2	14 (33%)	8 (33%)	6 (33%)	1.00
IFN-α	20 (48%)	11 (46%)	9 (50%)	0.79
GMCSF	13 (31%)	8 (33%)	5 (28%)	0.70
Anti-VEGF	8 (19%)	4 (17%)	4 (22%)	0.71
Vaccine	5 (12%)	4 (17%)	1 (6%)	0.37
Anti-BRAF	0	0	0	–
Anti-CTLA4	1 (2%)	1 (4%)	0	–
Anti-PD1	0	0	0	–
Subsequent therapy				
Metastasectomy	11 (26%)	4 (17%)	7 (39%)	0.16
Radiation Therapy (not brain)	9 (21%)	6 (25%)	3 (17%)	0.71
Brain Radiation Therapy	11 (26%)	6 (25%)	5 (28%)	1.00
Chemotherapy	19 (45%)	11 (46%)	8 (44%)	1.00
Interleukin-2	7 (8%	5 (21%)	2 (11%)	0.68
IFN-α	3 (7%)	2 (8%)	1 (6%)	1.00
GM-CSF	7 (17%)	4 (17%)	3 (17%)	1.00
Anti-VEGF	4 (10%)	2 (8%)	2 (11%)	1.00
Vaccine	1 (2%)	0	1 (6%)	–
Anti-BRAF	7 (17%)	3 (12%)	4 (22%)	0.44
Anti-CTLA4	12 (29%)	7 (29%)	5 (28%)	1.00
Anti-PD1	1 (2%)	0	1 (6%)	0.43
None	9 (21%)	7 (29%)	2 (11%)	0.26

*MACVAC* melanoma antigen cancer vaccine trial, *TCV* tumor cell vaccine, *DCV* dendritic cell vaccine, *IFN-α* interferon alpha, *GM-CSF* granulocyte macrophage colony stimulating factor, *VEGF* monoclonal antibody to vascular endothelial growth factor, *BRAF* enzyme endcoded by mutated BRAF gene, *CTLA4* cytotoxic T Lymphocyte Antigen 4, *PD1* programmed death molecule 1

### Study objectives

#### Efficacy

**Fig. 1:**
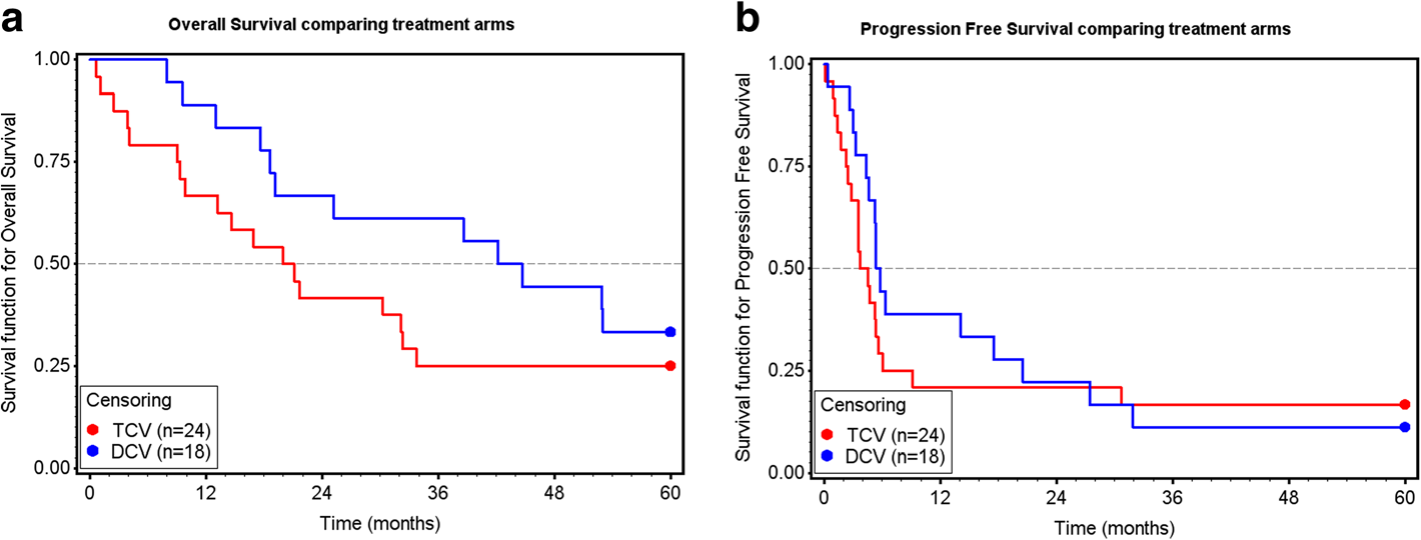
**a** Overall Survival by treatment arm. Median OS was 43.4 months versus 20.5 months for DCV and TCV respectively (18.6 to > 60 vs 9.3 to 32.3 months, 95% CI) (*p* = 0.194 Mantel-Haenzsel; *p* = 0.088 Gehan’s Wilcoxon). Adjusted Cox proportional hazard model revealed a 70% reduction in risk of death in the DCV arm (HR = 0.304, 95% CI 0.131 to 0.702, *p* = 0.0053, Wald test). Variables in multivariate analysis included age, stage, LDH, performance status, gender, M1 category, whether patient had measurable disease, treatment with dendritic cell vaccine, and whether patient lived outside California (see Additional file 4: Table S3. **b** Progression free survival (PFS) by treatment arm. Median PFS was 5.4 months in the DCV arm and 3.7 months in the TCV arm (4.0 to 8.0 vs 1.0 to 5.0 months, 95% CI *p* = 0.498)

DCV was associated with longer OS (Fig. 1a); there was no increase in PFS (Fig. 1b). A Cox regression analysis assessed the association between survival and other variables (Additional file 4: Table S3). Only DCV therapy and tumor burden (defined as measurable, detectable/equivocal but unmeasurable, or no evidence of disease) were strongly associated with survival. With the caveat that numbers of patients in subsets are quite small, for completeness survival by treatment arm for each of various clinical subsets associated with prognostic variables are shown in Table 3. In an earlier analysis at a time when minimal follow up was three years, and 17 patients were still in follow up, treatment in the DCV arm was the only variable associated with a survival difference. (Additional file 5: Table S4). As previously reported, there was one delayed complete response in a DCV-treated patient with progressing measurable disease when randomized [26]. At the time of 5-year follow up, she still had received no other anti-cancer therapy and was still in complete remission. Only one patient interrupted vaccine treatment to take another therapy, and then resumed the vaccine. That patient was in the TCV arm, stopped treatment to receive chemotherapy that he had received previously, and was still alive after 5 years.

**Table 3:** Treatment effects in various subsets

Subset	RX Arm	# Pts	Median OS Months	3-year OS	*P* value
Measurable	DCV	8	17.6	38%	0.122
	TCV	9	9.0	11%	
Not Measurable	DCV	10	44.6	80%	0.332
	TCV	15	32.2	33%	
Not NED	DCV	14	25.2	50%	0.011
	TCV	13	9.9	8%	
NED	DCV	4	53.0	75%	0.561
	TCV	11	33.7	40%	
High LDH St 4	DCV	6	17.6	17%	0.010
	TCV	3	1.1	0%	
WNL LDH	DCV	11	53.0	82%	0.079
	TCV	20	21.1	25%	
KPS =100	DCV	9	44.6	67%	0.810
	TCV	12	32.2	33%	
KPS < 100	DCV	9	38.6	56%	0.303
	TCV	12	9.0	17%	
Stage 4	DCV	16	38.6	56%	0.292
	TCV	17	16.9	24%	
Stage 3	DCV	2	> 60	100%	0.141
	TCV	7	32.2	29%	

*RX arm* treatment arm, *Pts* patients, *OS* overall survival, *DCV* dendritic cell vaccine, *TCV* = tumor cell vaccine, *NED* no evidence of disease, *LDH* serum lactate dehydrogenase, *St 4* stage 4, *KPS* Karnofsky Performance Status

#### Delayed type hypersensitivity skin test reactivity

Tumor DTH tests were neither prognostic for survival nor predictive of therapeutic benefit in either arm. There was no difference between treatment arms in baseline DTH tests, nor in conversion rate at week-4 from a negative to positive or weakly positive tumor DTH test (TCV 5/20 vs DCV 1/17, *p* = 0.19) (Additional file 6: Table S5), nor in the rates of ever having a positive tumor DTH test (TCV 6/22 vs DCV 2/22, *p* = 0.26).

#### Safety

Both vaccines were well-tolerated. All eight planned doses were administered to 67% and 54% of patients in the DCV and TCV arms respectively. All early discontinuations were due to disease progression; no patients discontinued treatment because of toxicity. The frequencies of AEs attributed to study agents are summarized in Table 4. As in previous trials the most common AEs were injection site reactions and flu-like symptoms and nearly all treatment-related toxicity was mild to moderate in severity [20, 21]. There was only one grade 3 AE, a severe headache that occurred after the 8th and final DCV injection. Toxicity grade was higher in the TCV arm with 71% of TCV patients experiencing grade 2 or higher AEs as opposed to only 16% of DCV-treated patients (17/24 vs 3/18, *p* = 0.0007) (Table 5).

**Table 4:** Summary by frequency of AEs of any severity, felt to possibly, likely, or almost certainly caused by injection of vaccines

Adverse Event	All (*N* = 42)	TCV (*n* = 24)	DCV (*n* = 18)	*P* value
Injection site reactions	28 (67%)	16 (67%)	12 (67%)	1.00
Flu-like symptoms	14 (33%)	9 (38%)	5 (28%)	0.742
Nausea	8 (19%)	5 (21%)	3 (17%)	1.00
Bone discomfort	7 (17%)	4 (17%)	3 (17%)	1.00
Headache	6 (14%)	3 (12%)	3 (17%)	1.00
Fatigue	5 (12%)	5 (21%)	0 (0%)	0.060
Chills	5 (12%)	1 (4%)	4 (22%)	0.146
Pruritus	3 (7%	2 (8%)	1 (6%)	1.00
Arthralgias	3 (7%)	1 (4%)	2 (11%)	0.567
Fever	3 (7%)	3 (12%)	0 (0%)	0.247
Rash	2 (5%)	2 (8%)	0 (0%)	0.498
Hives (urticarial)	2 (5%)	2 (8%)	0 (0%)	0.498
Shingles	2 (5%)	2 (8%)	0 (0%)	0.498
Myalgias	1 (2%)	0 (0%)	1 (6%)	0.429

*TCV* tumor cell vaccine, *DCV* dendritic cell vaccine

**Table 5:** Highest grade of adverse events (AEs) felt possibly, likely or almost certainly caused by injection of vaccine

Grade	All (*N* = 42)	TCV (*n* = 24)	DCV (*n* = 18)
Grade 0	6 (14%)	2 (8%)	4 (22%)
Grade 1	16 (38%)	5 (21%)	11 (61%)
Grade 2	19 (45%)	17 (71%)	2 (11%)
Grade 3	1 (2%)	0	1 (5%)
Grade 4	0	0	0

*TCV* tumor cell vaccine, *DCV* dendritic cell vaccine

### Serum markers

**Fig. 2:**
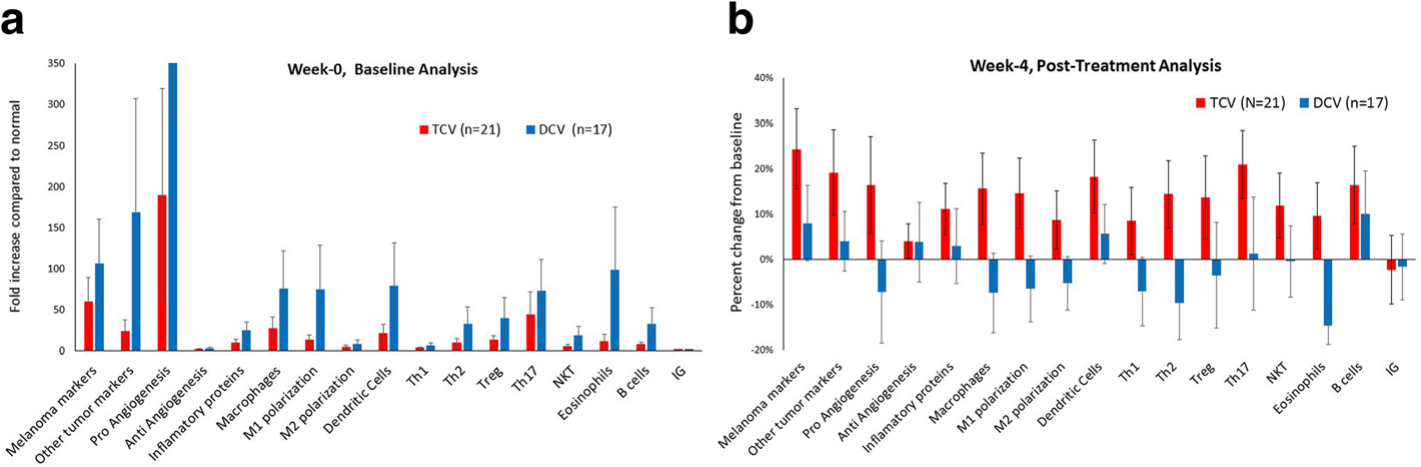
**a** Baseline analysis of serum cytokines. In order to summarize data for all tests, data is expressed as change in relation to values for set of assays compared to 3 normal control volunteers. There was substantial variation in markers among patients. At baseline most markers were elevated compared to normals, especially in the DCV arm. **b** The post treatment analysis of cytokines one week after the third weekly vaccine injection, showing changes compared to baseline. The changes associated with the two vaccines were quite different. Levels increased for nearly all markers in the TCV arm, but decreased for eight of the 17 groupings in the DCV arm

Paired week-0 and week-4 serum samples were available for 38 patients. Markers tested included 110 cytokines, growth factors, proteases, soluble receptors, and other proteins. Results were grouped together based on known associations with tumor growth, angiogenesis, and immune activation (Additional file 7: Table S6). At baseline serum levels of most markers were similar between the two arms, but tumor markers were higher in the DCV arm (Fig. 2), consistent with baseline tumor burden characteristics in that cohort (Table 1). The percent changes between week-0 and week-4 after the first three injections of DCV or TCV were quite different (Fig. 2b). TCV was associated with an increase in nearly all markers while DCV was associated with a decrease in several markers. In the TCV arm the tumor and inflammation markers increased from baseline. In the DCV arm tumor markers were only slightly increased from baseline, most inflammatory markers decreased from baseline, and some increased slightly. These results must be interpreted with caution because of the wide variability in assay results, and the use of only three donors for normalization.

## Discussion

To our knowledge, this is the first and only study conducted in cancer patients that addresses the question of whether survival differs between cancer patients treated with an autologous DCV versus an autologous TCV that both feature TAA from short-term autologous tumor cell lines. The most important observation in this study is that DCV was associated with a doubling of median OS and a 70% reduction in the risk of death compared to TCV. The median survival of 20.5 months in the TCV arm suggests that TCV may also have anti-tumor activity. The results in each arm of this randomized trial are quite similar to results previously reported for single arm trials of TCV [20], and DCV [21]. The lack of correlation between PFS and OS was also observed in the earlier trials, and has been observed for other immunotherapies that may provide long-lasting immune benefit [27]. Examples of FDA-approved agents that improved OS but had unimpressive ORR or PFS include sipuleucel-T in prostate cancer, [28] and ipilimumab in melanoma [29].

Strengths of the MACVAC trial include that it was a randomized trial, all patients received their assigned treatment, all patients were followed until death or for five years, and no patients were lost to follow up. Patients in both arms of the study were injected with vaccines that contained the patient-specific TAA from about 10 million tumor cells that were self-renewing in cell culture. The clinical study, including all leukapheresis procedures and treatment administration took place at a single institution, but patients were referred from all over the United States for this trial and remained under the clinical management of their referring physicians. Weaknesses of this trial are the small patient numbers, which was caused by the premature closure. Not only did this result in a much smaller population of subjects than originally planned, but also in an imbalance in the numbers of patients assigned to each treatment arm, and some imbalance in baseline prognostic factors. The imbalance in the treatment arms was due to the randomization of patients within each stratification, and the lack of blocking to assure approximately even randomization at any point in time. The imbalance in prognostic factors was slightly biased against the DCV arm. This also resulted in very small numbers of patients for exploratory subgroup analyses (Table 3), but this data was presented because of frequent questions from clinical other investigators regarding outcomes in these subgroups. Double-blinding was not used in this trial because it was a phase II trial, and because of the extra cost, inconvenience, and risks associated with leukapheresis procedures [30].

DTH tests to autologous tumor could be predictive of an effective anti-TAA immune response [31, 32]. We previously reported improved survival for 125 patients with various cancer histologies who had a positive DTH at any time during treatment with TCV [33], but this was not confirmed in TCV-treated metastatic melanoma patients [20]. There was no association between DTH reactivity and outcome in DCV-treated melanoma patients in the 54-patient single-arm trial [21]. The MACVAC trial confirmed that this tumor DTH test was not useful as a predictive or prognostic marker for either vaccine.

The MACVAC trial confirmed the limited toxicity associated with these vaccine products in previous trials [20, 21]. The most common AEs attributed to study injections were local injection site reactions and flu-like symptoms, which are well-known side effects of GM-CSF. Interestingly, there was somewhat higher grade toxicity (grade 2 as opposed to grade 1) in patients receiving TCV.

As part of this trial, serum was obtained at week-0 (baseline) and week-4 (after three weekly injections) and cryopreserved for later analysis. Comparisons of baseline samples and changes from week-0 to week-4 after the first three injections showed that the patterns of changes in cytokine levels differed between DCV and TCV. This data is included only to address the question raised by several investigators as to whether there was any evidence that there was a difference between the study arms in terms of changes in in any biological measurements after the first three weekly injections. Much more extensive evaluation would be needed to better understand the association between these changes and the clinical benefit observed in the DCV arm, especially in terms of immune effects on T cells and their recognition of tumor cells. Such experiments have not been performed because the cell samples obtained during this trial are no longer the property of the investigator.

The data regarding success in establishing tumor cell lines, and the time needed to establish them, is important for understanding the complex logistics involved in clinical trials of such patient-specific cellular products. Successful commercial application of autologous tumor cell lines would likely require a higher and faster rate of success in obtaining autologous tumor cells as a source of TAA in order to treat more patients, and to decrease potential differences in mutations between tumor cells growing in vitro and tumor cells growing in vivo. The objective of tissue culture was to increase the representation of self-renewing tumor cells (perhaps early progenitor cells) as the source of TAA rather than terminally differentiated tumor cells that predominate in a fresh tumor sample, However, this means that immune responses may be directed against only a small subset of cells in any tumor mass, which may explain why this approach is not associated with rapid regression of measurable tumor.

All attempts to establish dendritic cells were successful. In the DC arm no effort was made to standardize the number of DC injected, but the range of 3 million to 30 million cells among patients may be relatively narrow from a biological perspective. However, theoretically there could have been up to a 100-fold difference in the density of TAA per DC per patient. The potential impact of this is unclear given the autologous origin of the tumor cells and their unique neoantigens. Analyses failed to show any dose/survival relationship related to the number of dendritic cells injected among the 18 patients treated in this trial, and among the 54 reported previously [21].

Since MACVAC was conducted, enzyme-targeted BRAF inhibitors and monoclonal antibody immune checkpoint inhibitors, have become standard therapies for patients with metastatic melanoma, and are associated with improved survival [3, 7] Based on their complementary mechanisms of action, there is a good rationale for combining vaccines that present autologous TAA with these newer agents [34, 35]. There is also evidence that such patient-specific vaccines increase the survival benefit associated with the immune stimulating cytokine interleukin-2 [36]. Ex vivo loading of TAA onto DC outside of the immunosuppressive tumor environment may be especially advantageous in patients who have normal expression of major histocompatibility antigens, but have no infiltration of CD8+ T-lymphocytes in tumor biopsies. Pre-existing TAA recognition by the endogenous immune system is a prerequisite for possible benefit from anti-checkpoint therapy. DCV is worthy of further study combined or sequenced with these other agents. Other dendritic cell vaccines and patient-specific vaccine approaches already are showing promise in combination with checkpoint inhibitors [37, 38].

## Conclusions

This is the only clinical trial that has compared dendritic cell and whole tumor cell vaccines as platforms for autologous TAA presentation. DCV was associated with superior survival compared to TCV and induced different changes in serum cytokines than did TCV. DCV was associated with minimal toxicity. As a potential biomarker, DTH to autologous tumor cells was neither prognostic for survival nor predictive of benefit from DCV.

## Additional files

Additional file 1: **Table S1.** Success rate for cell cultures started from metastatic melanoma samples obtained during 2006–2011 (era of MACVAC trial). (DOCX 14 kb)

Additional file 2: **Table S2.** Relationship between stage at time of tumor collection for cell line and disease status at the time of randomization. (DOCX 13 kb)

Additional file 3: **Figure S1.** Consort diagram for MACVAC trial. (DOC 31 kb)

Additional file 4: **Table S3.** Mutlivariate Cox regression analysis and proportional hazards model for independent variables. (DOCX 14 kb)

Additional file 5: **Table S4.** Multiple univariate analyses performed when all patients were either deceased or had been followed for a minimum of three years. (DOCX 15 kb)

Additional file 6: **Table S5.** Results of delayed type hypersensitivity (DTH) skin test to autologous irradiated tumor cells, Week-0 (baseline) and Week-4 following three weekly vaccine injections. (DOCX 14 kb)

Additional file 7: **Table S6.** Panel of markers used to investigate the serum of patients treated in randomized phase II trial, grouped by associated biologic activity. (DOCX 15 kb)

## Abbreviations

AE: Adverse event
BRAF: Specific serine/threonine-protein kinase member of the Raf kinase family
CTLA-4: Cytotoxic T lymphocyte–associated antigen 4
DC: Dendritic cell
DCV: Dendritic cell vaccine
GM-CSF: Granulocyte-macrophage colony stimulating factor
ITC: Irradiated tumor cells
LDH: Lactate dehydrogenase
MEK: Mitogen-activated protein kinase
NED: No evidence of disease
OS: Overall survival
PD-1: Programmed cell death protein-1
PFS: Progression-free Survival
RECIST: Response criteria in solid tumors
SAE: Severe adverse event
TAA: Tumor associated antigens
TCV: Tumor cell vaccine
