# Supplementary material for: Randomized phase II trial of autologous dendritic cell vaccines versus autologous tumor cell vaccines in metastatic melanoma: 5-year follow up and additional analyses
Source: J Immunother Cancer. 2018 Mar 6;6:19. doi: 10.1186/s40425-018-0330-1 (PMC5840808; doi:10.1186/s40425-018-0330-1)
Supplement: Supplementary file 1 — Table S1. Success rate for cell cultures started from metastatic melanoma samples obtained during 2006–2011 (era of MACVAC trial). (DOCX 14 kb) [file 40425_2018_330_MOESM1_ESM.docx]

**Additional file 1: Table S1.** Success rate^*^ for cell cultures started from metastatic melanoma samples obtained during 2006-2011 (era of MACVAC trial)

| Size range in grams | # success | # samples | % success |
| --- | --- | --- | --- |
| All samples | 76 | 187 | 40.4% |
| 6.0 to 12.5 | 27 | 45 | 60% |
| 3.0 to < 6.0 | 19 | 34 | 56% |
| 2.0 to <3.0 | 7 | 25 | 28% |
| 1.0 to < 2.0 | 12 | 38 | 33% |
| 0.6 to < 1.0 | 8 | 22 | 36% |
| 0.2 to < 0.6 | 3 | 16 | 19% |
| <0.2 | 0 | 7 | 0% |
|  |  |  |  |
| > 3 gm | 46 | 79 | 58% |
| < 3 gm | 30 | 108 | 28% |

MACVAC =melanoma antigen cancer vaccine clinical

*Underestimates true success rate because denominator includes cultures that were discontinued for any reason, such as deterioration of patient. Of 76 samples, 42 were referred for treatment and all 42 were randomized and treated as randomized.

MD=measurable disease

NMD=non-measurable/equivocal disease

SRT=stereotactic radiation therapy

M1a= soft tissue metastases, normal LDH

M1b=lung metastases, normal LDH,

M1c= visceral disease or elevated LDH and M1a or M1b.
