# Supplementary material for: Randomized phase II trial of autologous dendritic cell vaccines versus autologous tumor cell vaccines in metastatic melanoma: 5-year follow up and additional analyses
Source: J Immunother Cancer. 2018 Mar 6;6:19. doi: 10.1186/s40425-018-0330-1 (PMC5840808; doi:10.1186/s40425-018-0330-1)
Supplement: Supplementary file 2 — Table S2. Relationship between stage at time of tumor collection for cell line and disease status at the time of randomization. (DOCX 13 kb) [file 40425_2018_330_MOESM2_ESM.docx]

**Additional file 2: Table S2** Relationship between stage at time of tumor collection for cell line and disease status at the time of randomization.

| Stage at Tumor Collection | # Pts | Status at RX | Progressive disease between tumor harvest and randomization |
| --- | --- | --- | --- |
| Regional recurrence | 18 | 9 NED  1 NED  1 NMD  7 MD | 9 No progression  1 M1c then, response to IL2-biochemo  1 M1b  1 M1a, 2M1b, 4 M1c |
| Distant metastasis | 24 | 5 NED  9 NMD  10 MD | 5 no additional therapy  2 no additional therapy (2 M1b)  1 GM-CSF (1 M1c)  2 biochemotherapy (2 M1c)  4 systemic + SRT brain mets (4M1c)  2 M1a, 4 M1b, 4 M1c |

NED=no evidence of disease

NMD=no measurable disease

MD=measurable disease
