# Supplementary material for: Randomized phase II trial of autologous dendritic cell vaccines versus autologous tumor cell vaccines in metastatic melanoma: 5-year follow up and additional analyses
Source: J Immunother Cancer. 2018 Mar 6;6:19. doi: 10.1186/s40425-018-0330-1 (PMC5840808; doi:10.1186/s40425-018-0330-1)
Supplement: Supplementary file 3 — Figure S1. Consort diagram for MACVAC trial. (DOC 31 kb) [file 40425_2018_330_MOESM3_ESM.doc]

**Additional file 3: Fig S1. Consort Diagram**

**Allocation**

**Analysis**

**Follow-Up**

**Enrollment, Stratification and Randomization**

Assessed for eligibility (n=42)

Excluded (n= 0)

♦  Not meeting inclusion criteria (n=0)

♦  Declined to participate (n= 0)

♦  Other reasons (n= 0)

Analysed (n= 24)
♦ Excluded from analysis (n= 0 )

Allocated to TCV (n=24)

♦ Received TCV (n= 24)

♦ Did not receive TCV (n= 0 )

Lost to follow-up (n= 0)

Discontinued DCV (Progressive Disease) (n= 6 )

Allocated to DCV (n= 18)

♦ Received DCV (n= 18)

♦ Did not receive DCV (n= 0)

Analysed (n= 18 )
♦ Excluded from analysis (n=0 )

Randomized (n= 42 )

LLost to follow-up (n= 0)

Discontinued TCV (Progressive Disease) (n= 11)
