# Supplementary material for: Randomized phase II trial of autologous dendritic cell vaccines versus autologous tumor cell vaccines in metastatic melanoma: 5-year follow up and additional analyses
Source: J Immunother Cancer. 2018 Mar 6;6:19. doi: 10.1186/s40425-018-0330-1 (PMC5840808; doi:10.1186/s40425-018-0330-1)
Supplement: Supplementary file 4 — Table S3. Mutlivariate Cox regression analysis and proportional hazards model for independent variables. (DOCX 14 kb) [file 40425_2018_330_MOESM4_ESM.docx]

**Additional file 4: Table S3.** Mutlivariate Cox regression analysis and proportional hazards model for independent variables.

| Parameter | Hazard Ratio | P value |
| --- | --- | --- |
| Age | 1.017 | 0.975 |
| Stage 4 | 0.967 | 0.958 |
| Elevated LDH | 0.924 | 0.919 |
| Live outside California | 1.589 | 0.226 |
| Karnofsky > 90 | 0.533 | 0.212 |
| Gender | 0.573 | 0.144 |
| M1 Category | 5.660 | 0.078 |
| Measurable Disease | 5.777 | 0.0035 |
| Dendritic Cell Vaccine | 0.304 | 0.0053 |

LDH=serum lactate dehydrogenase

M1=distant metastatic melanoma (categories M1a, M1b, or M1c)
