# Supplementary material for: Randomized phase II trial of autologous dendritic cell vaccines versus autologous tumor cell vaccines in metastatic melanoma: 5-year follow up and additional analyses
Source: J Immunother Cancer. 2018 Mar 6;6:19. doi: 10.1186/s40425-018-0330-1 (PMC5840808; doi:10.1186/s40425-018-0330-1)
Supplement: Supplementary file 5 — Table S4. Multiple univariate analyses performed when all patients were either deceased or had been followed for a minimum of three years. (DOCX 15 kb) [file 40425_2018_330_MOESM5_ESM.docx]

**Additional file 5: Table S4.** Results of univariate analyses performed at a time when all patients were either deceased or had been followed for a minimum of 3 months, with 17 patients still being followed. At that time point treatment in the DCV arm was the only variable that reached statistical significance (p<0.05)

| **Variable** | **OS < 3 yrs (n=25)** | **OS > 3 yrs (n=17)** | **P-value** |
| --- | --- | --- | --- |
| Age > 60 years | 11 (44%) | 6 (35%) | 0.57 |
| # Male | 15 (60%) | 12 (71%) | 0.48 |
| # from out of state | 8 (32%) | 8 (47%) | 0.21 |
| KPS = 100 | 11 (44%) | 10 (59%) | 0.12 |
| ↑LDH at randomization | 8 (32%) | 3(18%) | 0.48 |
| Highest stage =4 | 20 (80%) | 13 (76%) | 0.78 |
| Prior visceral &/or brain mets | 13 (52%) | 9 (53%) | 0.95 |
| Measurable Disease | 13 (52%) | 4 (24%) | 0.11 |
| NED at randomization | 6 (24%) | 9 (47%) | 0.055 |
| Stage 4 M1a at randomization | 1 (4%) | 2 (12%) | 0.56 |
| Stage 4 M1b at randomization | 6 (24%) | 3 (18%) | 0.72 |
| Stage 4 M1c at randomization | 12 (48%) | 3 (12%) | 0.056 |
| **DCV treatment** | 7 (28%) | 11 (65%) | **0.018** |

DCV=dendritic cell vaccine

KPS=Karnofsky performance status

NED=no evidence of disease

OS=overall survival
