# Supplementary material for: Randomized phase II trial of autologous dendritic cell vaccines versus autologous tumor cell vaccines in metastatic melanoma: 5-year follow up and additional analyses
Source: J Immunother Cancer. 2018 Mar 6;6:19. doi: 10.1186/s40425-018-0330-1 (PMC5840808; doi:10.1186/s40425-018-0330-1)
Supplement: Supplementary file 6 — Table S5. Results of delayed type hypersensitivity (DTH) skin test to autologous irradiated tumor cells, Week-0 (baseline) and Week-4 following three weekly vaccine injections. (DOCX 14 kb) [file 40425_2018_330_MOESM6_ESM.docx]

**Additional file 6: Table S5.** Results of delayed type hypersensitivity (DTH) skin test to autologous irradiated tumor cells, Week-0 (baseline) and Week-4 following three weekly vaccine injections.

| Week-0 – Week-4 | All (n=40) | TCV (n=22) | DCV (n=18) |
| --- | --- | --- | --- |
| Negative-Negative | 31 (78%) | 15 (68%) | 16 (89%) |
| Negative-weakly positive | 2 (5%) | 1 (4%) | 1 (5%) |
| Negative-Positive | 4 (10%) | 4 (18%) | 0 |
| Weakly positive –Negative | 1 (2%) | 1 (4%) | 0 |
| Weakly positive for both | 0 | 0 | 0 |
| Weakly positive –Positive | 0 | 0 | 0 |
| Positive-Negative | 0 | 0 | 0 |
| Positive-Weakly positive | 0 | 0 | 0 |
| Positive-Positive | 2 (5%) | 1 (4%) | 1 (5%) |

TCV=tumor cell vaccine; DCV=dendritic cell vaccine

There were two patients in the TCV arm who had negative baseline tumor cell DTH tests, and did not have a repeat tumor cell DTH at week 4, hence n=40 rather than 42, and TCV n=22 rather than 24.
