# Supplementary material for: Randomized phase II trial of autologous dendritic cell vaccines versus autologous tumor cell vaccines in metastatic melanoma: 5-year follow up and additional analyses
Source: J Immunother Cancer. 2018 Mar 6;6:19. doi: 10.1186/s40425-018-0330-1 (PMC5840808; doi:10.1186/s40425-018-0330-1)
Supplement: Supplementary file 7 — Table S6. Panel of markers used to investigate the serum of patients treated in randomized phase II trial, grouped by associated biologic activity. (DOCX 15 kb) [file 40425_2018_330_MOESM7_ESM.docx]

**Additional file 7: Table S6.** Panel of serum markers used to investigate changes associated with dendritic cell and tumor cell vaccines. Markers are organized by associated biological activity.

| Grouping | Subgroups | Markers |
| --- | --- | --- |
| Growth  factors | TGFb  Other | BigH3 (TGFb-induced),LAP,TGFb1,TGFb2,TGFb3  Activin A,bFGF,EGF,EGFR,HGFR,PDGFAA,PDGFAB,VEGF |
| Angiogenic  factors | Pro-Angio  VEGF Fam  Anti-angio | ANG1,Angiogenin,ANGPLT4  VEGF,FEGFC,FEGFD,VEGFR1,VEGFR2,VEGFR3  ANG2,Angiostatin |
| Tumor  markers | Melanoma  Other | GRO, GROa,S100b Galectin3, Ecadherin,Pcadherin  NSE,LDH,AFP,CA125,CA15-3,CA19-9,CEA,ErbB2,B2M,PD1 |
| Th1 | Modulating  Secreted | IL2,IL12,IL18, IL27,IFNg  IL2,IFNg,TNFa,TNFb,FASL |
| Th2 | Modulating  Secreted | IL2,IL4,IL6  IL1,IL3,IL4,IL5,IL6,IL10,IL13,IL25 |
| Th17 | Modulating  Secreted | TGFb1,IL1b,IL6,IL23,IL12p40  IL17,IL17F,IL21,IL22,TNFa |
| Treg | Modulating  Secreted | TGFb  TGFb,IL10,IL4 |
| NKT | Modulating  Secreted | IL2,IL12,IL15,IL18  IL17,IL22,MIP-1,RANTES,TNFa |
| Macrophages | Modulating  Secreted Chemotaxis  M1 stimulus  M1response  M2 stimulus  M2 response | GMCSF,MCSF  IL1a,IL1b,IL6,IL8,IL10,IL12p40,IL12p70,IL18,IL23,IL27,MCSF,Rantes,TNFa  MIF,GRO,GROa,VACM1  IFNg,GMCSF,IL1b,IL6,TNFIL8,GCSF,MCSF  IL15RA,IL2RA,IL6R,ICAM-1,IL6,IL8,GCSF,MCSF  IL4,IL13,MCSF  IL10,IL1R2,CD163,VEGF,TGFb1,IL1RA |
| Dendritic  Cells | Modulating  Secreted | GMCSF,IFNg,IL4  GMCSF,IL1a,IL1b,IL6,IL8,IL10,IL12,IL18,IL23,IL27,IP10, MCSF, RANTES, TGFb,TNFa |
| B cells | Modulating  Secreted | IL7,BAFF  IgA,IgD,IgE,IgG1,IgG2,IgG3,IgG4,IgM |
| Eosinophils | Modulating  Secreted | GMCSF,IL3,IL5,Eotaxin,RANTES,MCP1  IL1,IL2,IL4,IL5,IL6,IL8,IL13,TNFa |
| Inflammatory proteins | Acute Phase  Other | CRP,SAA  TNFRII,PAR,oncostatinM,gp130 |
| Tissue  remodeling |  | OPG,PAI1,TIMP1,TIMP2,MMPI,MMP2,MMP9 |
